# Clinical Grade of Obstetric Anal Sphincter Injuries and Prediction of Mode of Birth Recommendations: A 20‐Year Retrospective Analysis

**DOI:** 10.1111/1471-0528.18303

**Published:** 2025-07-22

**Authors:** Nicola Adanna Okeahialam, Ranee Thakar, Abdul H. Sultan

**Affiliations:** ^1^ St Mary's Hospital Manchester UK; ^2^ Brunel University London UK; ^3^ Croydon University Hospital Croydon UK; ^4^ St George's University of London London UK

**Keywords:** anal incontinence, anal manometry, anorectal physiology, endoanal ultrasound, obstetric anal sphincter injury

## Abstract

**Objective:**

To determine whether assessment of symptoms and clinical grade of obstetric anal sphincter injuries (OASIs) is predictive of subsequent endoanal ultrasound (EAUS) and anal manometry (AM) findings to guide mode of birth recommendations.

**Design:**

Twenty‐year retrospective analysis.

**Setting:**

Tertiary urogynaecology unit.

**Population or Sample:**

Women (*n* = 607) with a history of OASI in the second half of a subsequent pregnancy, 2002–2022.

**Methods:**

A St Mark's Incontinence Score (SMIS), AM and EAUS were completed. An elective caesarean section (ELCS) was recommended if there was an external anal sphincter (EAS) defect and an incremental maximum squeeze pressure (IMSP) < 20 mmHg.

**Main Outcome Measures:**

Accuracy, sensitivity, specificity, negative and positive predictive values (NPV and PPV) with 95% CI were calculated for the assessment of anorectal symptoms and clinical grade of tear relative to EAUS and AM findings.

**Results:**

Accuracy of symptom assessment and clinical grade of tear in determining those with an EAS defect and IMSP < 20 was 75.4% (95% CI 69.3%), 69.6% (95% CI 63.8%–75.0%), 62.7% (95% CI 50.0–74.2) and 43.6% (95% CI 27.8%–60.4%) with 3a, 3b, 3c and fourth degree tears, respectively. 3a tears had the highest NPV for EAS defect and IMSP < 20 (100.0% [95% CI 97.9–100.0]), EAS defect alone (97.1% [95% CI 94.7%–98.4%]) and IMSP < 20 alone (93.5% [95% CI 90.1–82.1]).

**Conclusions:**

Symptom assessment and clinical grade of OASI cannot be used solely to guide mode of delivery recommendations in a subsequent birth. Absence of symptoms in women with 3a tears has a high NPV, meaning these women can be recommended a vaginal birth.

## 1 Introduction

Obstetric anal sphincter injury (OASI) is a significant risk factor for the development of anal incontinence after vaginal birth [1]. Anal incontinence following OASI has been shown to affect 16% of women with a 3a tear, 18% with a 3b tear, 21% with a 3c tear and 28% of women with a fourth degree tear, 6 weeks to 5 years postpartum [2, 3]. In the National Health Service (NHS), between 2000 and 2011, OASI rates had increased from 1.8% to 5.9% [4]. In light of the rising trend in OASI rates in the United Kingdom, and the associated morbidity associated with anal incontinence, it is imperative that women are counselled appropriately in a subsequent pregnancy with regard to mode of a subsequent birth.

Endoanal ultrasound (EAUS) and anal manometry (AM) can be used to assess the integrity and function of the anal sphincter [5]. The Royal College of Obstetricians and Gynaecologists (RCOG) recommend that all women with OASI should be offered mode of birth counselling, which should include review of symptoms and use of EAUS or AM where available [6]. There are six modes of delivery protocols which use EAUS and AM published in the literature and there is great variation in the criteria for a vaginal birth or caesarean section recommendation [7, 8, 9, 10, 11, 12]. In addition, definitions of abnormal AM and EAUS differ across these protocols with caesarean section recommendations being made with the presence of abnormal EAUS and AM in combination or in isolation [7, 8, 9, 10, 11, 12]. However, the evidence to support planned caesarean section to prevent the development of new or worsening symptoms after OASI is heterogeneous and unclear [13].

Providing personalised care and support to women within maternity services is a priority initiative in the NHS, particularly with regards to OASI, in order to reduce long‐term morbidity [14]. Unfortunately, EAUS and AM are not available in all units. This was shown in a questionnaire‐based survey of all NHS hospitals in 2009, which found that only 32% of units had a dedicated perineal clinic with EAUS and AM [15]. Therefore, providing a personalised risk assessment and counselling women, particularly those that are asymptomatic, can prove difficult. Therefore, the aim of this study was to evaluate whether assessment of symptoms and the clinical OASI grade can be used to guide subsequent mode of birth recommendations, based on a standardised protocol.

## 2 Methods

Between November 2002 and March 2022, data from all women with a history of OASIs who attended the perineal clinic at the tertiary urogynaecology unit at Croydon University Hospital in a subsequent pregnancy were entered prospectively into the patient database. All the women were referred from within Croydon University Hospital or the surrounding regions. We follow a set protocol for the management of women in a subsequent pregnancy (https://www.perineum.net/information). Advice is given to women regarding subsequent mode of delivery on the basis of symptoms, EAUS, and AM findings alone. As the anonymous patient database is part of normal practise for the perineal clinic, institutional board and research ethics committee approval was not deemed necessary. Patient consent is obtained for inclusion in the database. This research received no specific grant from any funding agency in the public, commercial, or not‐for‐profit sectors. Patients and the public were not involved in this research study.

Women were reviewed in the second half of a subsequent pregnancy following the index delivery where the OASI occurred. Women with two OASIs were excluded. A St Mark's Incontinence Score (SMIS) was completed, followed by AM Stryker Pressure Monitor/ [16, 17, 18] Anopress device (*THD* Worldwide, Correggio [RE], Italy) [19] and EAUS both performed in a left lateral position, using the Pro‐focus 2202 or Flex‐focus 500 ultrasound system (BK Medical, Herlev, Denmark). A SMIS of ≥ 1 was considered symptomatic [8]. With AM, the difference between the maximum resting pressure and the maximum squeeze pressure; the incremental maximum squeeze pressure (IMSP) was calculated. The IMSP isolates the voluntary contribution of the external anal sphincter (EAS) to anal canal resting pressure and so directly correlates with EAS function [20]. Anal sphincter defect sizes were measured using a three‐point angle, with images taken at the deep (proximal), superficial (mid) and subcutaneous (distal) levels. A significant sphincter defect is defined as a discontinuity of greater than 30° in at least two‐thirds of the length of the anal sphincter [21]. A full‐thickness EAS defect of > 1 h (> 30°) in size and an IMSP of < 20 mmHg was considered abnormal.

Using our protocol, an elective caesarean section (ELCS) was recommended in a subsequent pregnancy if there was an EAS defect > 1 h and IMSP < 20 mmHg. A vaginal birth was recommended in both asymptomatic and symptomatic women if EAUS and AM were normal. The presence of a concomitant IAS defect is not taken into account for mode of delivery recommendation in our unit as we have previously found that in women with an IAS defect with an EAS scar or defect, subsequent birth does not affect anorectal function or symptoms [8]. However, subgroup analysis was performed to evaluate the effect of IAS defects as they are predictive of anal incontinence [22].

Data was analysed using SPSS. Nominal data is expressed as numbers and percentages. Continuous data is expressed as mean and standard deviation (SD). The independent Student's *t*‐test was performed to compare means with SDs of continuous data, and the relationship between categorical variables was evaluated using the Chi‐square or Fisher's exact test where appropriate. A corresponding *p* value of < 0.05 was considered statistically significant. Multinomial regression models were used to calculate odds ratios (ORs), adjusted OR and 95% confidence intervals (CI). Accuracy, sensitivity, specificity, negative and positive predictive values (NPV and PPV), negative likelihood ratios (LR−) and positive likelihood ratios (LR +) with 95% CI were calculated for presence of anorectal symptoms and clinical grade of tear relative to EAUS and AM findings as the primary reference standard among all participants.

## 3 Results

Overall, 1103 women were referred to the perineal clinic, of which 607 (55.0%) women had a graded OASI. Women were seen between 8 months to 12 years (mean = 3.83 years) following the index delivery where they sustained an OASI. Of the 607 women, 228 (37.6%) had a 3a, 273 (45.0%) had a 3b, 67 (11.0%) had a 3c, and 39 (6.4%) had a fourth degree tear. Four hundred twenty‐five women (70.0%) were asymptomatic and 182 (30.0%) were symptomatic. Using our protocol, ELCS was recommended in 15 (3.5%) asymptomatic and 11 (6.0%) symptomatic women. In comparison to asymptomatic women, symptomatic women with a history of OASI were older and significantly more likely to have a residual EAS defect and an IMSP < 20 mmHg. Also, there was a significant difference (*p* = 0.006) in the grade of OASI between symptomatic and asymptomatic women (Table 1).

**TABLE 1:** Maternal and perineal clinic investigation characteristics.

	Symptomatic (*n* = 182) Mean (SD)/*n*(%)	Asymptomatic (*n* = 425) Mean (SD)/*n*(%)	*p*
Age at time of review (years)	33.7 (9.7)	32.1 (4.6)	0.006^a^
Time between index delivery and review (months)	46.0 (106.5)	39.7 (23.0)	0.429^a^
BMI (m/kg^2^)	25.6 (4.8)	25.3 (5.0)	0.547^a^
Ethnicity			
White	109 (59.9)	243 (57.2)	0.911^b^
Asian	56 (30.8)	129 (30.4)	
Black	13 (7.1)	39 (9.2)	
Chinese	2 (1.1)	4 (0.9)	
Mixed ethnicity	0 (0.0)	2 (0.5)	
Other	2 (1.1)	8 (1.9)	
Parity			
1	112 (61.5)	262 (61.6)	0.667^c^
2	59 (32.4)	144 (33.9)	
> 2	11 (6.0)	19 (4.5)	
Tear Grade			
3a	58 (31.9)	170 (40.0)	0.006^c^
3b	79 (43.4)	194 (45.6)	
3c	25 (13.7)	42 (9.9)	
Fourth	20 (11.0)	19 (4.5)	
EAS defect			
Yes	44 (24.2)	45 (10.6)	< 0.001^c^
No	138 (75.8)	380 (89.4)	
IAS defect			
Yes	57 (31.3)	49 (11.5)	< 0.001^c^
No	125 (68.7)	376 (88.5)	
IMSP < 20 mmHg			
Yes	51 (28.0)	65 (15.3)	< 0.001^c^
No	131 (72.0)	360 (84.7)	
Any defect and IMSP < 20 mmHg			
Yes	19 (10.4)	21 (4.9)	0.012^c^
No	163 (89.6)	404 (95.1)	
Mode of delivery recommendation			
VB	171 (94.0)	410 (96.5)	0.161^c^
ELCS	11 (6.0)	15 (3.5)	

Abbreviations: ELCS, Elective caesarean section; *n*, Number; SD, Standard deviation; VB, Vaginal birth.

^a^ Student *t*‐test.

^b^ Fisher's Exact.

^c^ Chi‐Square.

A full‐thickness EAS defect was present in 10 (4.4%) 3a, 46 (16.8%) 3b, 14 (20.9%) 3c and 19 (48.7%) fourth degree tears. IMSP was abnormal in 27 (11.8%) 3a, 62 (22.7%) 3b, 17 (25.4%) 3c and 10 (25.6%) fourth degree tears. All variables had a *p* value < 0.05 on univariate analysis and were considered for multivariate logistic regression. In addition, based on our protocol, there was a significant difference (*p* = 0.002) in mode of delivery recommendation as an ELCS was recommended in 2 (0.9%) 3a, 14 (5.1%) 3b, 6 (9.0%) 3c and 4 (10.3%) fourth degree tears (Table 2).

**TABLE 2:** Univariate analysis of perineal clinic investigations.

Tear grade	IMSP < 20		EAS defect		Mode of delivery based on CUH protocol	
	Yes	No	Yes	No	VB	ELCS
3a	27 (11.8)	201 (88.2)	10 (4.4)	218 (95.6)	226 (99.1)	2 (0.9)
3b	62 (22.7)	211 (77.3)	46 (16.8)	227 (83.2)	259 (94.9)	14 (5.1)
3c	17 (25.4)	50 (74.6)	14 (20.9)	53 (79.1)	61 (91.0)	6 (9.0)
Fourth	10 (25.6)	29 (74.4)	19 (48.7)	20 (51.3)	35 (89.7)	4 (10.3)
*p*	< 0.005^a^		< 0.001^a^		0.002^b^	

Abbreviations: CUH, Croydon University Hospital; ELCS, Elective caesarean section; VB, Vaginal birth.

^a^ Chi‐Square.

^b^ Fisher's Exact.

In asymptomatic women, compared to 3a tears, an increased tear grade increased the odds of an IMSP < 20 mmHg (3b adjOR 3.32 [1.63–6.77], 3c adjOR 4.63 [1.83–11.77]). However, this was not significant with fourth degree tears (adjOR 1.48 [0.34–6.47]). In comparison to 3a tears, the odds of an EAS defect increased with tear grade. However, when IMSP < 20 mmHg was added to the regression model, this did not remain significant for 3c tears. In symptomatic women, the odds of an EAS defect increased with tear grade. There was no difference in IMSP (Table 3).

**TABLE 3:** Multivariate analysis of Perineal clinic investigations.

Asymptomatic				
	OR (95% CI)	*p*	adjOR (95% CI)^d^	*p*
IMSP < 20 mmHg^b^				
3b^a^	3.75 (1.86–7.58)	< 0.001	3.32 (1.63–6.77)	< 0.001
3c^a^	5.12 (2.04–12.87)	< 0.001	4.63 (1.83–11.77)	< 0.001
Fourth^a^	2.71 (0.68–10.73)	0.156	1.48 (0.34–6.47)	0.56
EAS defect^c^				
3b^a^	4.88 (1.83–13.05)	0.002	4.07 (1.50–11.06)	0.006
3c^a^	4.46 (1.23–16.19)	0.023	3.46 (0.93–12.91)	0.065
Fourth^a^	36.67 (10.34–130.03)	< 0.001	35.06 (9.77–125.84)	< 0.001
Symptomatic				
IMSP < 20 mmHg^b^				
3b^a^	1.03 (4.83–2.21)	0.973	1.09 (0.50–2.36)	0.828
3c^a^	0.83 (0.28–2.45)	0.734	0.92 (0.31–2.79)	0.887
Fourth^a^	1.41 (0.48–4.18)	0.532	1.64 (0.53–5.06)	0.393
EAS defect^c^				
3b^a^	3.83 (1.35–10.90)	0.012	3.92 (1.37–11.15)	0.01
3c^a^	5.96 (1.75–20.36)	0.004	5.92 (1.73–20.26)	0.005
Fourth^a^	8.67 (2.43–30.93)	< 0.001	8.89 (2.48–31.84)	< 0.001

Abbreviations: 95% CI, 95% confidence interval; adjOR, adjusted odds ratioOR, Odds ratio.

^a^ Reference standard =3a.

^b^ Reference standard = IMSP ≥ 20 mmHg.

^c^ Reference standard = No EAS defect.

^d^ adjusted for IMSP and EAS defect.

The diagnostic performance of assessing anorectal symptoms and clinical grade of tear relative to EAUS and AM findings as the primary reference standard is presented in Table 4. Assessing anorectal symptoms in combination with 3a demonstrated a 75.4% accuracy (95% CI 69.3%–80.9%), sensitivity of 100.0% (95 CI% 15.8–100.0) and specificity of 75.2% (95% CI 69.1–80.7) for diagnosing an EAS defect and IMSP < 20. 3b tears had 69.6% accuracy (95% CI 63.8–75.0), sensitivity of 35.7% (95 CI% 12.8–64.9) and specificity of 71.4% (95% CI 65.5–76.9). 3c tears had 62.7% accuracy (95% CI 50.0–74.2), sensitivity of 50.0% (95 CI% 11.8–88.2) and specificity of 63.9% (95% CI 50.6–75.8). Fourth degree tears had 43.6% accuracy (95% CI 27.8–60.4), sensitivity of 25.0% (95% CI 0.63–80.6) and specificity of 45.7% (95% CI 28.8–63.4). The PPV was poor among all grades of tear (3a 3.5% [95% CI 2.8–4.3]), 3b 6.3% [95% CI 3.2–12.3], 3c 12.0% [95% CI 5.4–24.5], fourth 5.0% [95% CI 0.9–22.8]). 3a tears had the highest NPV for all outcomes, including EAS defect and IMSP < 20 (100.0% [95% CI 97.9–100.0]), EAS defect alone (97.1% [95% CI 94.7–98.4]) and IMSP < 20 alone (93.5% [95% CI 82.1–90.1]).

**TABLE 4:** Diagnostic performance of the assessment of anorectal symptoms with grade of OASI relative to EAUS and AM results.

Grade of tear	Statistic	EAS defect + IMSP < 20% (95% CI)	EAS defect alone% (95% CI)	IMSP < 20 alone% (95% CI)
3a	Accuracy	75.4 (69.3–80.9)	74.56 (68.4–80.1)	76.8 (70.7–82.1)
	Sensitivity	100.0 (15.8–100.0)	50.0 (18.7–81.3)	59.3 (38.8–77.6)
	Specificity	75.2 (69.1–80.7)	75.7 (69.4–81.2)	79.1 (72.8–84.5)
	PPV	3.5 (2.8–4.3)	8.6 (4.6–15.5)	27.6 (20.1–36.5)
	NPV	100.0 (97.9–100.0)	97.1 (94.7–98.4)	93.5 (82.1–90.1)
	LR+	4.0 (3.2–5.0)	2.1 (1.1–4.0)	2.8 (1.9–4.3)
	LR−	0.0 (0.0–0.0)	0.7 (0.4–1.2)	0.5 (0.3–0.8)
3b	Accuracy	69.6 (63.8–75.0)	69.6 (63.8–75.0)	64.5 (58.5–70.1)
	Sensitivity	35.7 (12.8–64.9)	45.7 (30.9–61.0)	35.5 (23.7–48.7)
	Specificity	71.4 (65.5–76.9)	74.4 (68.3–80.0)	73.0 (66.5–78.9)
	PPV	6.3 (3.2–12.3)	26.6 (19.8–34.8)	27.8 (20.5–36.6)
	NPV	95.4 (93.3–96.8)	74.4 (83.7–90.0)	79.4 (75.9–70.1)
	LR+	1.3 (0.6–2.6)	1.8 (1.2–2.6)	1.4 (0.9–2.2)
	LR−	0.9 (0.6–1.3)	0.7 (0.6–1.0)	0.9 (0.8–1.1)
3c	Accuracy	62.7 (50.0–74.2)	68.7 (56.2–79.4)	55.2 (42.9–67.4)
	Sensitivity	50.0 (11.8–88.2)	64.3 (35.1–87.2)	35.3 (14.2–61.7)
	Specificity	63.9 (50.6–75.8)	69.8 (55.7–81.7)	62.0 (47.2–75.4)
	PPV	12.0 (5.4–24.5)	36.0 (24.2–49.8)	24.0 (13.2–39.7)
	NPV	92.9 (85.1–96.7)	88.1 (78.2–93.9)	73.8 (65.1–81.0)
	LR+	1.4 (0.6–3.3)	2.1 (1.2–3.8)	0.9 (0.5–1.9)
	LR−	0.8 (0.3–1.8)	0.5 (0.3–1.0)	1.0 (0.7–1.6)
Fourth	Accuracy	43.6 (27.8–60.4)	46.2 (30.1–62.8)	59.0 (42.1–74.4)
	Sensitivity	25.0 (0.63–80.6)	47.4 (24.5–71.1)	70.0 (34.8–93.3)
	Specificity	45.7 (28.8–63.4)	45.0 (23.1–68.5)	55.2 (35.7–73.6)
	PPV	5.0 (0.9–22.8)	45.0 (30.6–60.3)	35.0 (23.3–48.8)
	NPV	84.2 (73.2–91.3)	47.4 (32.1–63.2)	84.2 66.2–93.6)
	LR+	0.5 (0.1–2.6)	0.9 (0.5–1.6)	1.6 (0.9–2.8)
	LR−	1.6 (0.8–3.2)	1.2 (0.6–2.2)	0.5 (0.2–1.5)

Abbreviations: LR +, Positive likelihood rationLR−, Negative likelihood ratio; NPV, Negative predictive value; PPV, Positive predictive value.

Subgroup analysis of IAS defects showed that this was seen on EAUS in 14 (6.1%) 3a, 45 (16.5%) 3b, 20 (29.9%) 3c and 27 (69.2%) fourth degree tears (Table S1). Overall, 11.8% (*n* = 59) of women with a 3a/b tear had a residual IAS defect. In those with a 3a tear, 4.1% (*n* = 7) of asymptomatic and 12.1% (*n* = 7) of symptomatic women had an IAS defect. In those with a 3b tear 11.9% (*n* = 23) of asymptomatic and 27.8% (*n* = 22) of symptomatic women had an IAS defect (Table S2). Significantly more symptomatic women had an IAS defect in comparison to asymptomatic women (31.3% [*n*=57/182] vs. 11.5% [n=49/425], *p* < 0.001). This remained significant in those with a 3a/b tear and a residual IAS defect (21.2% [*n* = 29/137] vs. 8.2% [*n* = 30/364], *p* < 0.001).

## 4 Discussion

### 4.1 Main Findings

This original study found that the assessment of symptoms and the clinical grade of OASI is not useful in guiding subsequent mode of birth recommendations. However, our findings suggest that the absence of symptoms with 3a tears has a 94%–100% NPV with respect to EAUS and AM results, meaning these women can be recommended a vaginal birth.

### 4.2 Strengths and Limitations

The main strength of this study is the large cohort of patients from a prospectively collected database over a 20‐year period, which minimises the risk of recall bias and selection bias. Additionally, the use of the gold standard imaging modlaity [5] to assess anal sphincter integrity, a standardised protocol [8] and validated tools such as the SMIS [23] to assess anal sphincter function strengthen our findings further. Limitations firstly include that in our unit had to replace the Stryker Pressure Monitor with the Anopress device in 2017, because the catheter was no longer available, meaning 278 (45.8%) and 329 (54.2%) women respectively underwent AM with different devices. Although these devices have been validated, it is known that catheter design and sensor configuration can cause minor differences in pressure measurement, which is unlikely to bias the results of this study significantly [24]. Also, as AM and EAUS were not performed or analysed blind to OASI grade, this introduces potential measurement bias. Unfortunately, as our unit is a tertiary referral centre, only 55% of women were referred with a documented grade of OASI. However, this was unlikely to change our findings as misclassification of OASI can occur, which has an impact on anal sphincter function and therefore symptoms. This is particularly with regard to the involvement of the IAS, as endosonographic evidence of an IAS defect, identified 3 months following primary repair of OASIs, has been shown to increase the odds of faecal incontinence five‐fold [22]. In our study, 11.8% of 3c tears were misdiagnosed as a 3a/3b tear. Additionally, in comparison to those correctly diagnosed (no IAS defect), an additional 13% had a SMIS ≥ 1. This is similar to the retrospective cohort study by O'Leary et al. [25] of 615 women where IAS defects were seen in 9.1% with a 3a/3b tear and these women also had a significantly higher Wexner score. It has been shown that anal sphincter injuries assessed immediately postpartum and then repeated 7 weeks later, are not ‘occult’ but missed tears of the anal sphincter, that can be identified by a proper vaginal and rectal examination immediately after delivery and before any suturing [26]. EAUS has been validated histologically [27, 28] and shown to have a 100% and 96% specificity for detecting EAS and IAS defects [29], which highlights that misdiagnosis can occur.

## 5 Interpretation

This is the first study to demonstrate that the assessment of symptoms and the clinical grade of OASI is not useful in guiding subsequent mode of birth recommendations. This is because the grade of the initial tear does not predict the presence of residual defects or impaired function; instead, it is the post‐repair anatomy and physiology that guide birth recommendations. This requires correct clinical diagnosis and adequate primary repair. Sideris et al. [30] demonstrated in their systematic review of 16 110 women that following primary repair of OASI, 55% of women had a persistent sphincter defect and by follow‐up at less than 3 months and over 1 year, 34% and 45%, respectively, will experience anal incontinence. Although at our mean follow‐up period of 4 years, 30% were symptomatic, Mous et al. [31] demonstrated in their 25‐year longitudinal study of 171 women with OASI that symptom prevalence increased from 38% to 61%. Moreover, in asymptomatic women, by 15 and 25‐year follow‐up 43% and 11%, respectively, had developed symptoms. Therefore, early assessment of symptoms alone may be inadequate as symptom prevalence increases with time. Anal incontinence aetiology is multifactorial and women with OASI may not present with incontinence until later on in life due to age‐related changes in sphincter function [32, 33]. However, symptoms can occur with an intact sphincter due to stretching of the pudendal nerve during vaginal birth, secondary to factors such as multiparity, forceps, prolonged second stage and increased fetal birth weight [32, 34].

The RCOG recommend that in a subsequent birth following OASIs, an ELCS should be offered to women who are symptomatic or have abnormal EAUS or AM findings [6]. Although we demonstrated that in symptomatic women, the odds of an EAS defect significantly increased with the grade of tear, the assessment of symptoms in combination with the clinical grade of OASI had suboptimal diagnostic accuracy with regards to EAUS and AM findings. Therefore, when EAUS or AM cannot be offered, there is the potential for women who are symptomatic, with no anal sphincter trauma/an EAS scar alone to undergo an ELCS when their symptoms are not secondary to anal sphincter integrity compromise. Moreover, women with symptoms secondary to an isolated IAS defect may also be recommended an ELCS. However, a vaginal birth can be considered, with no significant worsening of symptoms or change in resting pressure, which reflects the function of the IAS [8]. This emphasises the value of these investigations in the follow up of women with OASI. As caesarean section may not be protective against anal incontinence following OASI [13] including anal incontinence in the long‐term [35], appropriate counselling is essential to allow women to make a fully informed decision about a subsequent mode of birth.

With asymptomatic women, we found that the odds of an EAS defect significantly increased with the grade of tear and the odds of an IMSP < 20 mmHg increased particularly with 3a tears in comparison to 3b and 3c. EAUS and AM are not readily available in all hospitals. Additionally, EAUS is expensive and requires a trained operator to perform the investigation and accurately interpret the images [36]. The use of AM in combination with EAUS is useful, as the IMSP has been shown to have good diagnostic accuracy with regards to anal incontinence severity [37]. Although in asymptomatic individuals there is no evidence directly correlating abnormal AM with symptom development, AM results in women with residual sphincter defects have been shown to reduce significantly at 10‐year follow‐up [38]. As AM evaluates sphincter function, which is a key factor in maintaining continence, abnormal findings in asymptomatic women are a marker of anal sphincter compromise, especially in the presence of a sphincter defect. Reid et al. [39] have shown that these women are at increased risk of developing symptoms over time. Approximately two‐thirds of women with an anal sphincter defect may be asymptomatic; therefore, there is significant benefit in providing both EAUS and AM to aid counselling of asymptomatic women with regards to mode of birth in a subsequent pregnancy [30, 40]. However, as our study showed that the absence of symptoms in women with 3a tears has a 94%–100% negative predictive value with regards to EAUS and AM results, this will be useful information in this cohort of women, particularly in low‐resource settings, where these specialist investigations are not available.

## 6 Conclusion

Mode of delivery recommendations in a subsequent birth following OASI cannot be made based on the presence of symptoms and clinical grade of tear alone. This study highlights the need for EAUS and AM to allow both symptomatic and asymptomatic women to make a fully informed decision about a subsequent mode of birth.

## Author Contributions

N.A.O.: Project conception and design. Data extraction, analysis, interpretation. Drafting of manuscript and final approval of version to be published. Agreement to be accountable for all aspects of the work in ensuring that questions related to the accuracy or integrity of any part of the work are appropriately investigated and resolved. R.T.: Project conception and design. Data interpretation. Critical review of draft and final approval of version to be published. Agreement to be accountable for all aspects of the work in ensuring that questions related to the accuracy or integrity of any part of the work are appropriately investigated and resolved. A.H.S.: Project conception and design. Data interpretation. Critical review of draft and final approval of version to be published. Agreement to be accountable for all aspects of the work in ensuring that questions related to the accuracy or integrity of any part of the work are appropriately investigated and resolved.

## Ethics Statement

Research ethics committee approval was not required.

## Conflicts of Interest

The authors declare no conflicts of interest.

## Supporting information


Tables S1–S2.

## Data Availability

The data that support the findings of this study are available from the corresponding author upon reasonable request.
