# Supplementary material for: Clinical Grade of Obstetric Anal Sphincter Injuries and Prediction of Mode of Birth Recommendations: A 20‐Year Retrospective Analysis
Source: BJOG. 2025 Jul 22;132(12):1802–9. doi: 10.1111/1471-0528.18303 (PMC12501750; doi:10.1111/1471-0528.18303)
Supplement: Supplementary file 1 — Tables S1–S2. [file BJO-132-1802-s001.docx]

**Appendix**

**Table S1: Univariate analysis of perineal clinic investigations**

| **Tear grade** | **IAS defect** | |
| --- | --- | --- |
|  | **Yes** | **No** |
| **3a** | 14(6.1) | 214(93.9) |
| **3b** | 45(16.5) | 228(83.5) |
| **3c** | 20(29.9) | 47(70.1) |
| **4th** | 27(69.2) | 12(30.8) |
| **p-value** | <0.001* | |

**Table S2: Univariate analysis of symptoms following IAS injury based on grade of OASI**

| **Asymptomatic** | | | |
| --- | --- | --- | --- |
| **Tear Grade** | **IAS defect** | **No IAS defect** | **p-value** |
| **3a** | 7 (4.1) | 163 (95.9) | ***<0.001*** |
| **3b** | 23 (11.9) | 171 (88.1) |  |
| **3c** | 8 (19.0) | 34 (81.0) |  |
| **4^th^** | 11 (57.9) | 8 (42.1) |  |
| **Symptomatic** | | | |
| **3a** | 7 (12.1) | 51 (87.9) | ***<0.001*** |
| **3b** | 22 (27.8) | 57 (72.2) |  |
| **3c** | 12 (48.0) | 13 (52.0) |  |
| **4^th^** | 16 (80.0) | 4 (20.0) |  |
